# Policy *v*. practice: school food practices do not reflect healthy food guidance in New Zealand primary schools

**DOI:** 10.1017/S1368980025101341

**Published:** 2025-10-20

**Authors:** Danika Pillay, Ajmol Ali, Carol Wham

**Affiliations:** School of Sport, Exercise and Nutrition, Massey University, Auckland, New Zealand

**Keywords:** Food policy, Menu audit, Policy evaluation, Traffic-light guidance, Healthy Active Learning, Childhood nutrition, School food environment

## Abstract

**Objective:**

To examine how school food policies and perceived barriers influence food provision in New Zealand primary school canteens, using the ‘Healthy Food and Drink Guidance for Schools’.

**Design:**

Cross-sectional analyses of school food menus and school food policy and practices surveys completed by school leaders/principals.

**Setting:**

New Zealand primary schools.

**Participants:**

239 primary schools completed the school food policies and practices survey, and eighty schools provided canteen menus.

**Results:**

Most schools reported having a healthy food and drink policy in their school (76·2 %) and promoted healthy eating during school hours (87·4 %). Two-thirds (69·5 %) identified barriers to healthy food and drink provision, most commonly the convenience of ready-made foods (39·3 %), and resistance from parents (34·3 %). The number of reported barriers was not a significant predictor for the presence of a school food policy (OR-1·034, *P* = 0·841). School menus (*n* 80) consisted of 16·4 % ‘green’ items, 34·7 % ‘amber’ items and 36·8 % ‘red’ items. There was no relationship between the percentage of ‘green’, ‘amber’ and ‘red’ items and the presence of a school food policy or reported barriers. More than a third (38·9 %) of menus from schools that reported they had a ‘Plain Milk and Water’ only policy still contained sugar-sweetened beverages.

**Conclusions:**

Although most New Zealand primary schools had healthy food policies, this was not consistently reflected in healthy food items on canteen menus. Further research is needed to understand how systemic barriers, such as cost, convenience and parental influence, affect policy implementation and school food provision.

Unhealthy dietary behaviours are a contributing factor to childhood overweight and obesity, and poorer educational outcomes, health status and mental health among children and adolescents^(1)^. Dietary behaviours that develop during childhood and adolescence often continue into adulthood and may have significant impacts on the economic welfare and capacity of the healthcare system^(2,3)^. Obesogenic food environments have been identified as a major contributor to unhealthy dietary patterns^(4,5)^. The school food environment has been recognised as a significant setting for influencing dietary behaviours and has the potential to reach the majority of children and adolescents during key periods of growth and development^(6)^. The ‘Nutrition-Friendly Schools Initiative Framework’ developed by the WHO recognises that having comprehensive school nutrition policies incorporating multiple components of diet, physical activity, education and environmental changes can positively influence weight, diet and health behaviours in schoolchildren^(1)^.

Children attend school in New Zealand (NZ) for 6–7 h, 5 days a week from the age of 5 years^(7)^. Most children consume one-third of their daily food intake during school hours, and it is estimated that the average child will consume approximately 2400 lunches at school through their schooling years^(8,9)^. The NZ school food system is complex and encompasses packed lunches, school canteens and tuckshops, commercial catering, charitable donations, and for some, the Healthy School Lunches programme^(10)^. While the Healthy School Lunches programme (Ka Ora Ka Ako) in NZ was designed to provide free lunches to 25 % of students most in need based on the schools’ calculated equity index (EQI) score^(11)^, school canteens operate as facilities on site that students can purchase lunch or snacks from which also serves as source of income for the school^(12)^. Buying lunch from the school canteen has been associated with a higher intake of discretionary food, sugary drinks, and lower fruit and vegetable intake compared with packed lunches and food consumed at home^(13,14)^. The NZ government recently launched the Healthy Active Learning (HAL) initiative as part of its Child and Youth Well-Being Strategy in 2020^(15)^. In conjunction, the Ministry of Health released the voluntary ‘Healthy Food and Drink Guidance for Schools’ in 2020 to help schools create policies promoting healthy food and drinks within schools, replacing the ‘Food and Beverage Classification System’ previously used^(16)^. The guidance utilises a traffic-light system which classifies foods into ‘green’ (nutrient-rich), ‘amber’ (some nutritional value) and ‘red’ foods (low nutritional value, higher in saturated fat, salt or sugar). All schools are encouraged to ensure over 75 % of foods provided are in the ‘green’ category and that ‘red’ foods are not available.

Within the NZ Education (School Boards) Amendment Regulations 2022, the schools’ Board of Trustees must promote healthy food and nutrition to students, and schools can develop their own policies and processes for healthy food promotion^(7)^. School food policies have the potential to have positive impacts on the availability of nutritious food and drinks within schools^(1,17)^. The Ministry of Health developed a ‘Healthy Food and Drink Toolkit’ alongside the ‘Healthy Food and Drink Guidance for Schools’ to support schools to create personalised school food policies in conjunction with students, staff and the wider community^(18)^. School food policies provide a framework to improve the nutritional value of foods available within the school as well as promote healthy eating practices and nutrition education^(18)^. However, it has been shown that school nutrition policies in NZ are often weak and lack comprehensiveness due to the use of generalised food policy templates which lack individualisation, with poor adherence to healthy food and drink guidelines across schools^(19–22)^. Recent cross-sectional analyses have revealed that NZ school canteens predominantly offer unhealthy food items, such as sugar-sweetened beverages, pies and pastries, and highly processed sweet and savoury foods^(21,23,24)^. Both in NZ and internationally, multiple barriers to implementing healthy food and drink policies have been identified, including lack of policy implementation support and training, resistance from the school community, and the cost and convenience of pre-packaged and convenience foods.^(12,21)^.

Few studies have explored the influence of school food policies and perceived barriers on food availability in NZ school canteens^(21)^. This study aimed to assess whether the presence of school food policies and perceived barriers affect food provision within primary school canteens in NZ using the ‘Healthy Food and Drink Guidance for Schools’.

## Methods

### Healthy Active Learning

HAL is a joint government initiative between Sport NZ, the Ministry of Education, the Ministry of Health and Health NZ that supports schools, kura (Māori immersion schools^(25)^) and early learning services to improve the wellbeing of children and young people through healthy eating and drinking, and quality physical activity and curriculum delivery^(15)^.

Massey University has been contracted to evaluate the outcomes of the HAL initiative. The evaluation is a longitudinal quasi-experimental mixed methods design which began in 2020.

### Data collection and participants: school food policies and practices

**Figure 1:**
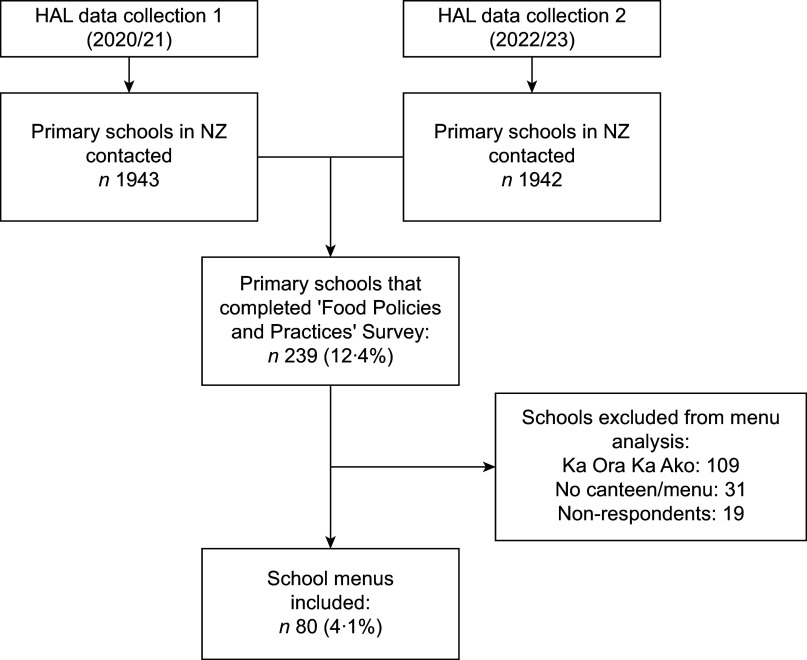
Data collection and participants completing the ‘Food Policies and Practices’ survey and providing school menus for analysis. Percentages represent the proportion of all NZ primary schools included in this analysis. HAL, Healthy Active Learning.

Surveys, adapted and developed by the Massey University research team, were used to capture information about the healthy food environments in schools. ‘School Food Policy and Practices’ surveys were sent out via email to all schools between 2020–2021 and 2022–2023, in a phased data collection period (Figure 1). Schools were identified via the website ‘Education Counts’ operated by the Ministry of Education^(26)^. To maximise participation across schools, email reminders were sent on four separate occasions, and responses were captured using Qualtrics software. These surveys were intended to be completed by school principals or a member of the senior management team. The surveys included whether the school had a formal, written healthy food and drink policy, policy communication and content, and school practices that promote a healthy eating environment based on the Ministry of Health guidance for schools. A total of 239 schools completed the Food Policies and Practices survey between 2020 and 2023.

Several demographic characteristics were collected to provide context for the study. School deciles, area (urban/rural), school size (number of students) and EQI were obtained from the NZ Ministry of Education School Directory. Decile ratings (1–10) were previously used by the Ministry of Education between 1995 and 2022 to assess how many students lived in low socio-economic communities. The lower the schools’ decile rating, the more funding it received from the Ministry of Education. The decile system was replaced by the EQI in 2023 due to a greater understanding of factors influencing student learning and which utilises a wider range of variables, taking into account more than just the residential neighbourhood where students reside^(27)^. Both the EQI and the decile school ratings were included to allow comparison to earlier literature. The schools’ deprivation level was determined using the 2023 NZ deprivation index (NZDep). The NZDep is an area-based measure of socio-economic deprivation in NZ based on several consensus variables where high scores correspond to higher levels of socio-economic deprivation. Schools’ decile ratings and NZDep scores were classified as low (1–3), medium (4–6) or high (7–10). EQI was reported per the Ministry of Education socio-economic reporting bands for 2024: low (344–428), moderate (429–493) or high (494–569)^(28)^. School size was classified as small (< 199), medium (200–399) or large (> 400) based on the number of students.

### Data collection and participants: school menus

Schools that completed the ‘School Food Policies and Practices’ questionnaire were approached by the primary researcher between 2020 and 2023 to request a copy of their school canteen menu. Of the 239 schools that completed the ‘Food Policies and Practices’ survey, 109 schools were excluded due to their participation in Ka Ora Ka Ako (‘Healthy School Lunches’ programme by the Ministry of Education) which likely altered their food provision systems beyond the school’s control. There were a further thirty-one schools excluded who had no canteen or food menu, and nineteen schools that did not respond to the request for their menu. Overall, a total of eighty schools provided menus included in the present menu analysis (Figure 1).

### Menu analysis

A team of four researchers (three nutritionists and one dietitian) developed a quick menu audit tool in 2021, termed the ‘Menu Analysis Toolkit’ to assess all menus collected in the HAL evaluation. Details of the toolkit, including development and categorisation of food items, have been reported previously^(23)^. In summary, the toolkit offers a detailed breakdown of commonly packaged foods and meals/main menu items available to purchase in schools, categorised according to a traffic-light system based on the Ministry of Health ‘Healthy Food and Drink Guidance for Schools’. The traffic-light system classifies school menu items as ‘green’ (nutrient-rich), ‘amber’ (some nutritional value) and ‘red’ (low nutritional value, higher in saturated fat, salt or sugar). A registered dietitian completed the school menu analysis using the ‘Menu Analysis Toolkit’, with 10 % of the menus assessed by a second dietitian to ensure consistency in food classification.

### Statistical analysis

Descriptive analyses were conducted for demographic variables, healthy food and drink policies in schools, and school practices that promote a healthy eating environment. Chi-square tests of independence were used to examine the relationship between school characteristics (type, region, decile, EQI, deprivation, school size and area) and the presence of a food and drink policy, as well as the relationship between water and milk only policies and the presence of sugar-sweetened beverages. Normality was tested using the Kolmogorov–Smirnov and Shapiro–Wilk tests, which indicated that the data for the percentage of ‘green’, ‘amber’ and ‘red’ foods were not normally distributed. Consequently, the Mann–Whitney *U* test was used to determine differences in the percentage of food items (‘green’, ‘amber’ and ‘red’) between schools with food policies and those reporting barriers. A binary logistic regression model was used to investigate whether the number of reported barriers predicted the presence of a food policy. All tests were two-tailed, and a *P*-value < 0·05 was considered significant.

## Results

A total of 239 schools were included for analyses. Schools were evenly split between contributing (49 %) and full primary (51 %), with most located in the Upper North Island (33·1 %) and urban areas (67·8 %) (see online supplementary material, Supplemental Table A). Most schools stated that they had a healthy food and drink policy in their school (76·2 %), while 5·4 % of schools reported that they did not have a food policy but would like to have one (Table 1). Sixty-four schools provided details on their school food policy, of which fifty-three reported their policy was on the website ‘SchoolDocs’^(29)^. ‘Plain milk and water only’ was stipulated by 52·7 % of schools, while 28·9 % did not make the distinction. Schools stated that the policy was likely to be utilised by the Board of Trustees (55·2 %), followed by whānau (family; 49 %), with food providers and canteen providers less likely to use it (22·6 % and 5 %, respectively). Healthy food and drink policies were mainly communicated via the school newsletter (68·2 %) and at staff meetings (53·6 %). Most were able to source the new Ministry of Health ‘Healthy Food and Drink Guidance for Schools’ from either the Ministry of Health (30·5 %) or Ministry of Education (35·6 %) website, but 16·3 % of schools were not aware of the new guidance (Table 1).

**Table 1.** Healthy food and drink policies in schools

		Total sample (*n* 239)	
		*N*	%
Presence of a healthy food and drink policy	Yes	182	76·2
	No but would like to	13	5·4
	No	30	12·6
Policy stipulates water and plain milk only	Yes	126	52·7
	No	69	28·9
Who is likely to utilise the policy^*^	Board of trustees	132	55·2
	Whānau (family)	117	49·0
	Students	104	43·5
	PTA	62	25·9
	Food providers	54	22·6
	Canteen providers	12	5·0
How is the policy communicated^*^	School newsletter	163	68·2
	Staff meetings	128	53·6
	School website	108	45·2
	Student assemblies	96	40·2
	Email to staff, students and others	61	25·5
	Information seminars/workshops	12	5·0
Awareness of *‘Healthy Food and Drink Guidance for Schools’*	Sourced from MoE website	85	35·6
	Sources from MoH website	73	30·5
	Not aware of guidelines	39	16·3

MoH, Ministry of Health; MoE, Ministry of Education; PTA, Parent–Teacher Association.

Some schools did not provide answers to certain questions.

* Multichoice answers.

Most (87·4 %) schools promoted healthy eating during school hours (Table 2). Fundraising support was reported by 15·1 % of schools using products from food and beverage companies such as Cadbury/Whitakers chocolate, Bakers Delights, Cookie Time cookies, pie providers and Pita Pit. More than half of schools (59 %) agreed or strongly agreed that their school had sufficient water fountains, while 22·2 % of schools either disagreed or strongly disagreed with this statement. External charitable support for food provision was reportedly received by 60·3 % of schools, with Kickstart Breakfast (44·8 %) and KidsCan (34·7 %) the most common initiatives.

**Table 2.** Health-promoting school practices

		Total sample (*n* 239)	
		*N*	%
Promotion of healthy eating during school hours	Yes	209	87·4
	No	3	1·3
Fundraising support by food and beverage companies^†^	Yes	36	15·1
	No	177	74·1
Sufficient water fountains in the school	Agree/strongly agree	141	59
	Disagree/strongly disagree	53	22·2
	Neither agree/disagree	27	11·3
Participation in an external programme	Yes	144	60·3
	No	76	31·8
External programmes^*^	Kickstart breakfast	107	44·8
	KidsCan	83	34·7
	Fonterra milk in schools	80	33·5
	Free school lunches (Ka Ora Ka Ako)	71	29·7
	Fruit in schools	69	28·9
	Community Breakfast Club	23	9·6
	Eat My Lunch	11	4·6

Some schools did not provide answers to all questions.

* Multichoice answers.

† Examples of fundraising support by food and beverage companies included Cadbury/Whitakers chocolate, Bakers Delights, Cookie Time cookies, Pita Pit, pie providers (Oxford/Fairlie/Naked Baker).

**Figure 2:**
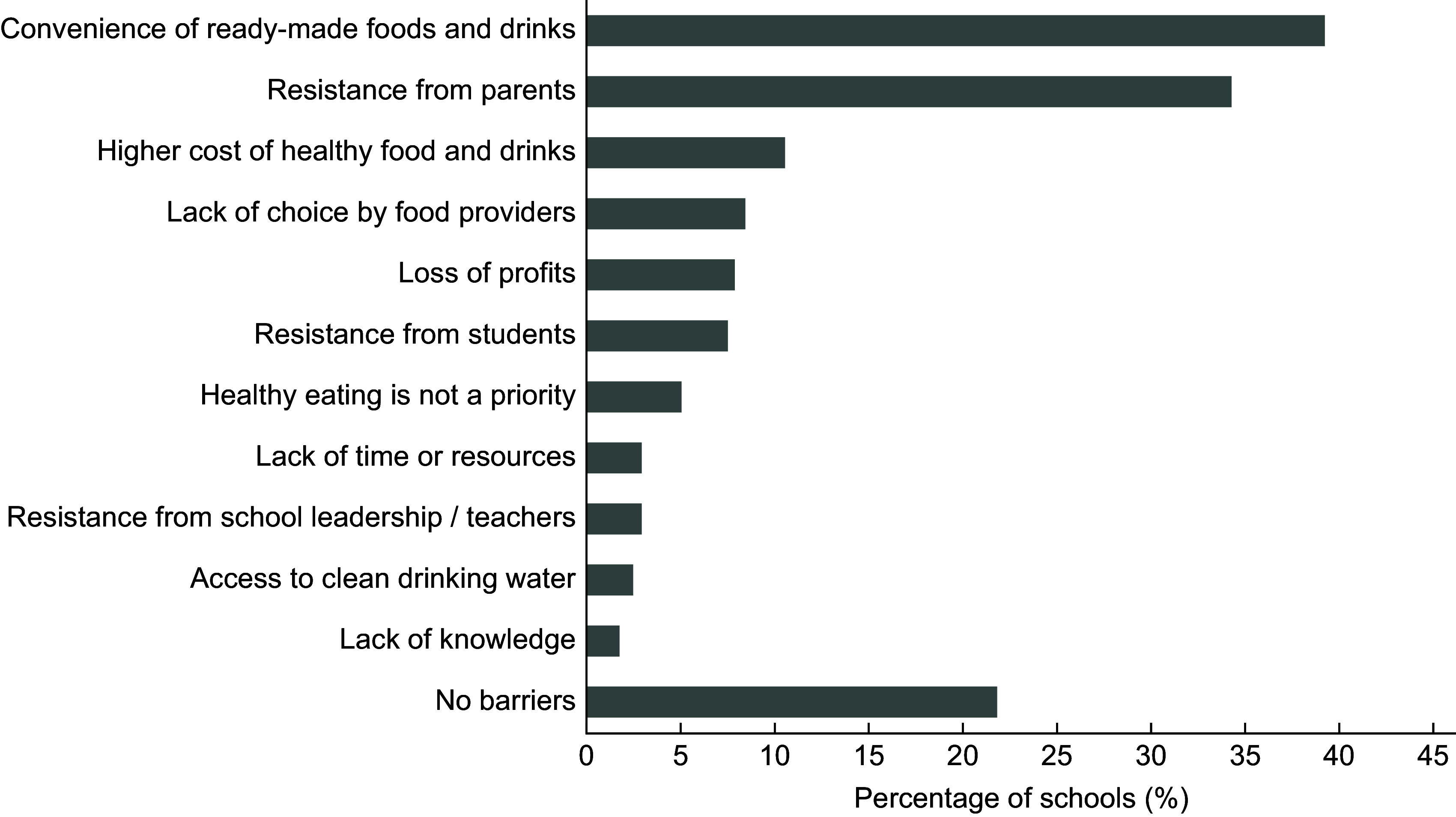
Barriers to healthy food and drink provision in schools. Multiple response answers. A total of 166 schools reported at least one barrier.

There were no significant differences between school characteristics (deciles, EQI, deprivation, size, area, type and region) and the presence of a healthy food and drink policy (*P* > 0·05; see online supplementary material, Supplemental Table A). Two-thirds (69·5 %) of schools reported a barrier to healthy food and drink provision, the most common being the convenience of ready-made food and drinks (39·3 %), followed by resistance from parents (34·3 %; Figure 2). Approximately 21·8 % of schools reported no barriers to healthy food and drink provision. The number of reported barriers was not a significant predictor of the presence of a school food policy (OR-1·034, *P* = 0·841).

### Menu analysis

**Figure 3:**
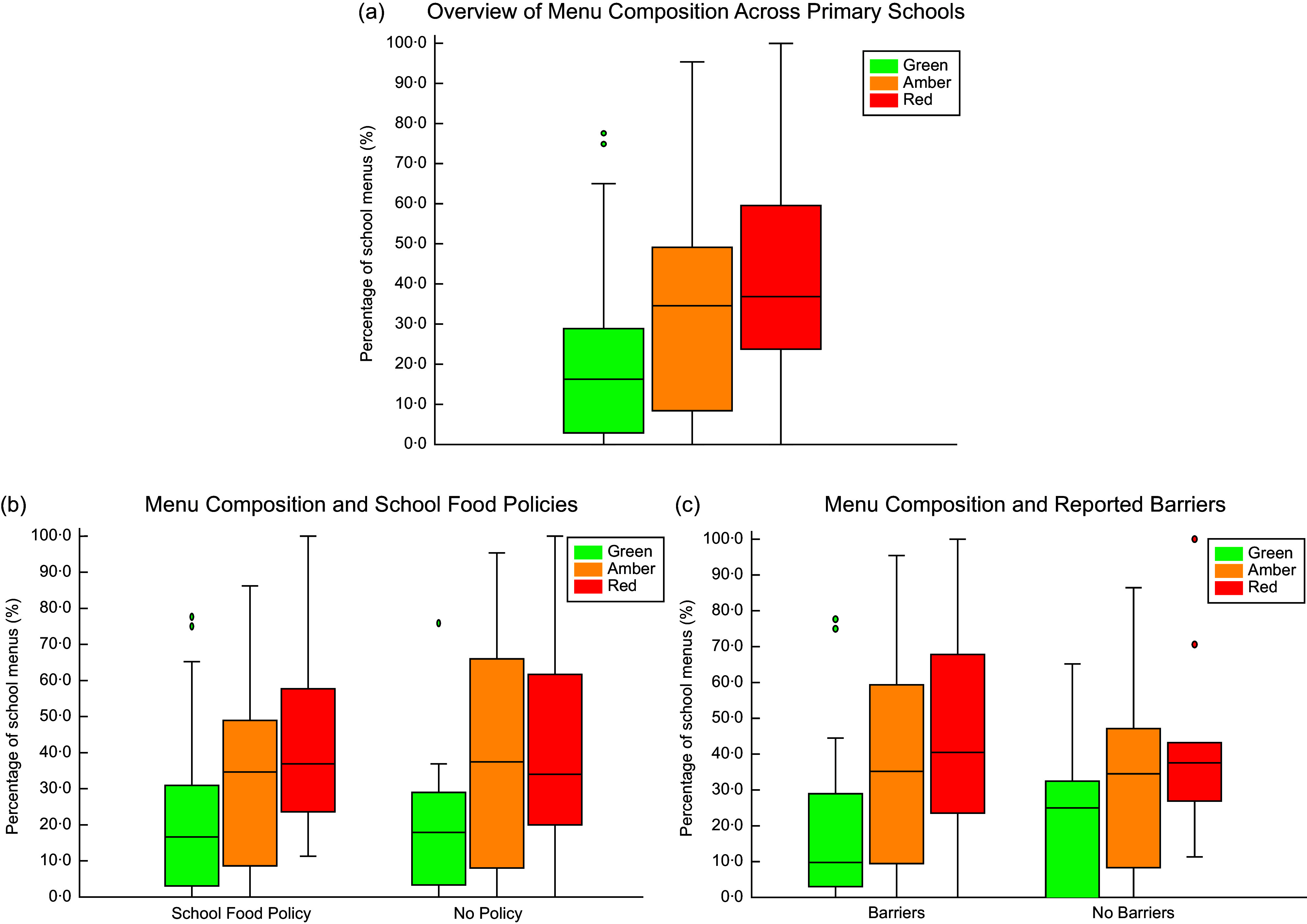
Healthiness of school food menus based on the presence of a Healthy Food and Drink Policy or perceived barriers. Values reported as median (25th, 75th percentile). Percentages of ‘green’, ‘amber’ and ‘red’ food items according to (a) total menu sample, *n* 80 schools, (b) presence of a school food policy and (c) reported barriers to healthy food and drink provision. The ‘No Policy’ category includes those who would like to have a food policy but currently do not. A total of 51/80 schools listed at least one barrier to providing healthy food and drinks in schools and are represented in the ‘Barrier’ category.

A total of eighty schools provided their food menus for analyses. The overall composition of these school menus comprised 16·4 % of ‘green’ items, 34·7 % ‘amber’ items and 36·8 % ‘red’ items (Figure 3(a); see online supplementary material, Supplemental Table B). There were no significant differences in the percentages of ‘green’ (U = 466, *P* = 0·858), ‘amber’ (U = 446·5, *P* = 0·668) and ‘red’ (U = 439·5, *P* = 0·605) food items between schools that had a healthy food and drink policy and those that did not (Figure 3(b)).

‘Green’ food items were lower in schools that reported barriers (9·7 %) compared to schools that did not (25 %); however, this did not reach statistical significance likely due to the small sample size and variation in within the groups (U = 428·5, *P* = 0·457). There were no differences in ‘amber’ (U = 459·5, *P* = 0·741) and ‘red’ (U = 480·5, *P* = 0·958) foods between schools that reported barriers and those that reported no barriers to implementing a healthy food and drink policy (Figure 3(c)).

**Figure 4:**
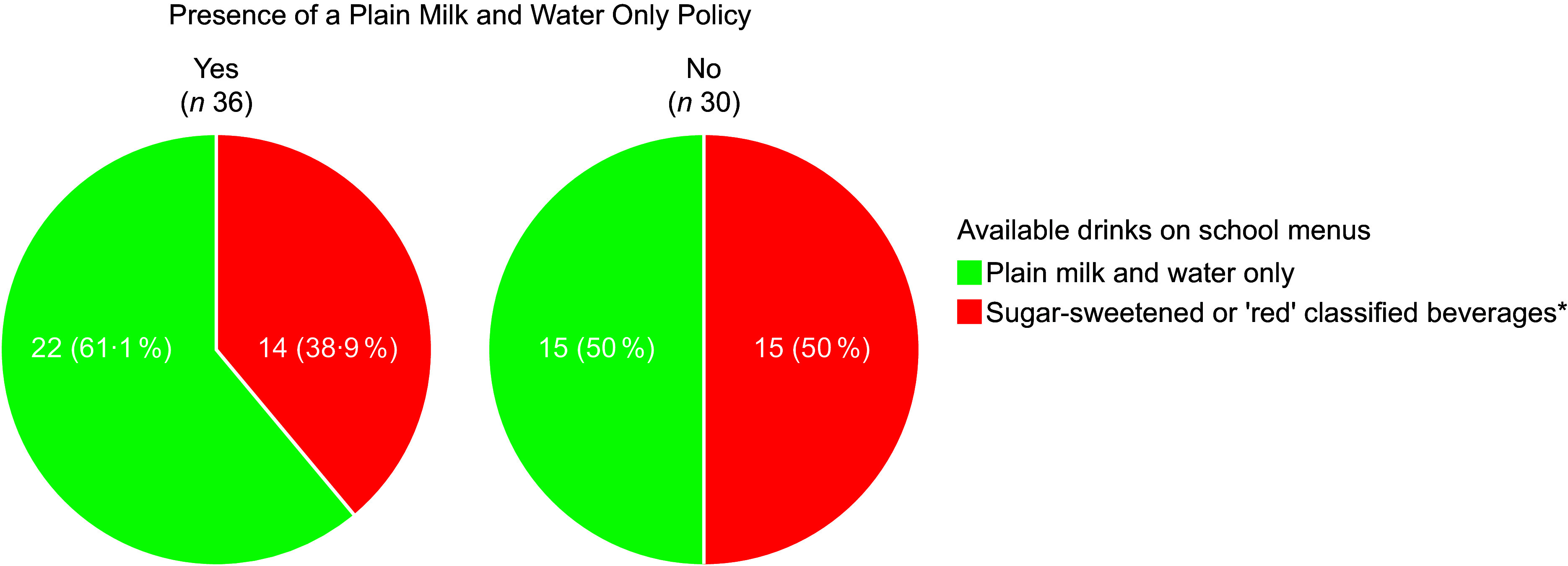
‘Plain Milk and Water Only’ policies and the availability of sugar-sweetened or ‘red’ classified beverages on school menus. *‘Red’ classification as per the Ministry of Health ‘Healthy Food and Drink Guidance for Schools’ in New Zealand which includes sugar-sweetened beverages, smoothies, milk-based drinks with added sugar, fruit/vegetable juices (including those with no added sugar), energy drinks and sports drinks, and flavoured water.

Almost half of schools (42·5 %) had sugar-sweetened or ‘red’ classified beverages, per the ‘Healthy Food and Drink Guidance for Schools’, available on their school food menus. The majority of sugar-sweetened or ‘red’ classified beverages available on school menus were juice/flavoured water (30 %), followed by flavoured milk (16·3 %) and other beverages including popsicles, frozen milk desserts and smoothies/blended fruit drinks (17·5 %). Exclusive water and plain milk were present in 52·5 % of school food menus. There was no association between having a ‘Plain Milk and Water’ only policy and the presence or absence of sugar-sweetened or ‘red’ classified beverages (χ^2^(1) = 0·820, *P* = 0·365). More than a third (38·9 %) of menus from schools that stated they had a ‘Plain Milk and Water’ only policy still contained sugar-sweetened or ‘red’ classified beverages (Figure 4).

## Discussion

This study highlights the persistent challenges in improving the nutritional quality of food menus in NZ primary school canteens. While most schools reported having a healthy food and drink policy (76·2 %) and many promoted healthy eating (87·4 %), this was not reflected in the food offered on school menus. Most school food menus were made up of unhealthy ‘red’ foods (36·8 %), followed by ‘amber’ foods (34·7 %), with healthy ‘green’ foods making up the smallest proportion (16·4 %). The lack of association between reported barriers and school food policies on food provision suggest that broader systemic factors such as school resources, government support and prioritisation by schools may influence food provision more than just policy existence. Implementing healthy food policies is often low on the list of school priorities when it comes to available resources and time, educational priorities and socio-economic barriers^(12,30)^. It has been reported that negative attitudes of school staff, limited sense of responsibility for students dietary choices and disagreement among staff members is a hindrance towards healthy food and drink policies^(12)^. However, adequate funding, effective policy communication and management, and positive attitudes have been reported as facilitators of policy implementation^(12)^.

Alongside the ‘Healthy Food and Drink Guidance for Schools’, many schools used a generic template from the website ‘SchoolDocs’ for their food and nutrition policies. While these templates have previously been shown to offer good comprehensiveness, their limited strength from using suggestive instead of prescriptive language, may reduce their overall effectiveness^(21,31)^. This is highlighted by the 38·9 % of schools who stated having a ‘plain milk and water only’ policy yet still offered sugar-sweetened or ‘red’ classified beverages on the school menu. School food policies need to be tailored to the specific needs of each school, as individualised approaches are a crucial factor in shaping the school food environment and ensuring meaningful impact^(31)^. Additionally, the voluntary nature of the ‘Healthy Food and Drink Guidance for Schools’ means that without proactive direction from staff or parents, food environments are often deprioritised and fail to adequately support students in making healthy dietary choices^(32)^. There has been variable uptake of similar government school guidelines across several Australian territories with few schools eliminating ‘red’/‘banned’ food items from school canteens despite guidelines being mandated^(33–36)^. However, Western Australian schools have shown greater compliance, with 89 % of schools meeting the nutritional criteria (> 60 % ‘green’ items, < 40 % ‘amber items’ and no ‘red’ items)^(37)^. This is likely due to the presence of clear and quantifiable targets, as well as the requirement for school principals to assess and report on the healthiness of their school canteen each year, highlighting that enforcement is critical for policy adoption and implementation^(19,37,38)^.

Multi-component interventions which integrate leadership support and engagement, staff training, tools and resources, and monitoring and feedback has been shown to improve the implementation of healthy food and drink policies within schools^(19,38–40)^. While the ‘Healthy Food and Drink Guidance for Schools’ remains voluntary, schools will need support to implement and prioritise healthy food and drink policies. The Theoretical Domains Framework (TDF), comprising of fourteen domains, offers a valuable lens for understanding the behavioural factors that influence policy implementation and adherence^(41)^. In Canada, the TDF was used to systematically examine barriers and facilitators affecting teachers’ implementation of the mandated Daily Physical Activity policy, with environmental context and resources, beliefs about consequences, social influences, knowledge and intentions emerging as key domains to inform targeted interventions and policy refinement^(42)^. Similarly, in Australia, the TDF was used to assess perceived barriers and enablers to dietary guideline implementation among early childhood education centre cooks, revealing that social/professional role and identity, beliefs about consequences, and skills were influential domains, with the skills domain significantly associated with greater menu compliance^(43)^. These findings highlight the value of theory-informed approaches in identifying implementation challenges and informing the design of more effective, context-specific interventions^(41)^.

Consistent with previous findings, the most common barriers to healthy food and drink provision were the convenience of ready-made foods (39·3 %) and resistance from parents (34·3 %). Low cost and convenience of ready-made foods are often cited as a barrier to healthy food provision^(12,21,32)^; however, a recent analysis of Australian lunchboxes, comparable to the NZ setting and economy, highlighted that lunchboxes made up of only healthy food items cost AUD$1·53 less than lunchboxes made up of > 50 % unhealthy foods on average^(44)^. Similarly, a randomised controlled trial of the implementation of a healthy canteen policy in Australia highlighted that there were no adverse financial effects of a healthier menu on canteen revenue^(40)^. Further research is needed to evaluate the financial implications of providing healthier food and drink options within the NZ school canteen setting. Interestingly, school principals and leaders reported that food and canteen providers were the least likely to utilise a healthy food and drink policy which may reflect limited opportunities for providers to contribute to policy decisions. Addressing barriers like cost and convenience through subsidies or partnerships with healthier food suppliers may help overcome some challenges. Increasing the prices of unhealthy food items to subsidise the cost of healthy foods could help balance the higher costs of healthier options while also discouraging student purchases of less nutritious choices^(31)^. However, further investigation is warranted to assess potential unintended consequences such as increased purchasing of unhealthy foods from external food retailers around schools.

As shown in the last children’s nutrition survey in NZ (2002), more than half of children bought some of the food consumed at school from the school canteen; however the majority (84·4 %) brought most of their food from home^(45)^. There has been limited data reported on the usage of school canteens in NZ in the last 20 years^(45,46)^. However, more recent data from Australia reflects a similar trend, with 84 % of surveyed parents (*n* 359) indicating that their children brought packed lunches to school every day^(47)^. Further studies are needed to investigate the extent to which students use school canteens and how this has changed over time in NZ. Schools’ perceptions of parental support for food policies may be influenced by implicit biases based on school lunch boxes^(47,48)^ potentially leading to misunderstandings about parental priorities. Recent cross-sectional quantitative and qualitative studies in Australia investigating parent perspectives on school food provision found that healthy and balanced meals were the most important factor for parents with regard to school food provision^(49,50)^. It is widely recognised that parents and students should be consulted in the development of school food policies and programmes^(10,18,51)^; however, parents and students in NZ have reported a lack of communication from schools with regard to changes to the food system and food policies^(48)^. Students benefit from lunch systems that involve them in menu planning and support autonomy, while parents emphasise the importance of non-stigmatising approaches to food that promote positive relationships with eating^(48,49)^. Engaging students and parents in the restructuring of school canteens to align with school food policies may help address schools’ concerns while ensuring that parental priorities as key stakeholders are acknowledged and integrated^(48,49)^.

### Strengths and limitations

This study represents the first analysis of the impact of school food policies and reported barriers on primary school canteen menus in NZ, using the ‘Healthy Food and Drink Guidance for Schools’. This study utilised a canteen menu audit tool, with menu coding completed by the primary researcher and dietitian, with reliability testing conducted by a second dietitian familiar with the dataset and menu analysis toolkit, ensuring consistency and accuracy in classification. Additionally, the study utilises a well-established traffic-light categorisation system to assess the nutritional value of foods, similar to Australia. This enhances comparability across Australia and NZ, which have similar school food provision models, allowing for broader insights into school food environments.

There are several limitations that must be considered when interpreting the results of the present study. Data collection for school menus took place in 2023/2024, whereas school food policies and practices questionnaires were collected in 2020/2021 and others in 2022/2023 as part of the structure of the HAL evaluation. This difference in timing means that any changes in school policies or priorities over the years may not have been fully captured. Additionally, the comprehensiveness of food policies was not assessed, limiting the ability to examine how detailed policies influence school menus. Although 239 schools completed the food policies and practices survey, the menu analyses included only eighty schools, which may not provide a fully representative view of school food environments. Future studies with a larger sample of school food menus may better highlight barriers and enablers to healthy food provision. Both the surveys and menus relied on self-reported data, introducing the possibility of bias and incomplete responses based on who was responsible for completing the survey. Social desirability bias may have influenced responses, leading some schools to overstate their adherence to healthy food guidelines and practices.

### Conclusions

This study highlights the challenges in implementing and maintaining healthy food and drink policies in NZ primary schools. While most schools report having healthy food and drink policies, inconsistencies in school practices and the prioritisation of food environments mean that these policies are not effectively reflected on school canteen menus. Standardised policy templates, while useful, lack the strength needed for meaningful implementation. The dominance of ‘red’ food items on menus suggests that systemic barriers, including cost and convenience, parental support and stakeholder engagement, may play a more significant role in food provision than policy existence alone. Implementing multicomponent interventions which include monitoring and feedback measures may help to ensure compliance with healthy food policies, particularly while the ‘Healthy Food and Drink Guidance for Schools’ remains voluntary.

## Supporting information

Pillay et al. supplementary material Pillay et al. supplementary material

## Supplementary material

For supplementary material accompanying this paper visit https://doi.org/10.1017/S1368980025101341
