# Supplementary material for: Policy v. practice: school food practices do not reflect healthy food guidance in New Zealand primary schools
Source: Public Health Nutr. 2025 Oct 20;28(1):e184. doi: 10.1017/S1368980025101341 (PMC12722081; doi:10.1017/S1368980025101341)
Supplement: Pillay et al. supplementary material [file S1368980025101341sup001.docx]

**Supplementary Files**

**Supplementary Table A:** Presence of a Healthy Food and Drink Policy based on school characteristics.

|  |  | Total sample (n=239)  n (%) | Schools with a Food and Drink Policy  n (% group) |
| --- | --- | --- | --- |
| Years^¥^ | Contributing (1-6) | 117 (49) | 89 (76.1) |
|  | Full primary (1-8) | 122 (51) | 93 (76.2) |
| Region^#^ | Upper North Island | 79 (33.1) | 56 70.9) |
|  | Central North Island | 48 (20.1) | 37 (77.1) |
|  | Lower North Island | 45 (18.8) | 36 (80.0) |
|  | Upper South Island | 37 (15.5) | 33 89.2) |
|  | Lower South Island | 30 (12.6) | 20 (66.7) |
| Decile | Low | 105 (43.9) | 82 (78.1) |
|  | Medium | 86 (36.0) | 68 (79.1) |
|  | High | 48 (20.1) | 32 (66.7) |
| Equity Index^^^ | Low | 50 (20.9) | 36 (72.0) |
|  | Medium | 108 (45.2) | 77 (71.3) |
|  | High | 81 (33.9) | 69 (85.2) |
| Deprivation | Low | 35 (14.6) | 27 (77.1) |
|  | Medium | 101 (42.3) | 76 (75.2) |
|  | High | 103 (43.1) | 79 (76.7) |
| School Size | Small | 124 (51.9) | 93 (75.0) |
|  | Medium | 71 (29.7) | 55 (77.5) |
|  | Large | 44 (18.4) | 34 (77.3) |
| Area | Urban | 162 (67.8) | 126 (77.8) |
|  | Rural | 77 (32.2) | 56 (72.7) |

¥ Contributing primary schools are defined as those teaching years 1-6 (ages 5-11). Full primary schools teach years 1-8 (ages 5-13).
# Regions have been condensed to accommodate for smaller sample sizes in some regions. Upper North Island combined Auckland and Northland; Central North Island includes Hawkes Bay, Waikato, Taranaki, and Bay of Plenty; Lower North Island includes Manawatu-Whanganui and Wellington; Upper South Island includes Marlborough, Canterbury, and West Coast; and Lower South Island includes Otago and Southland.
^^^Equity index reported per the Ministry of Education socioeconomic reporting bands for 2024: Low (344-428), moderate (429-493), high (494-569).
*p-value <0.05 considered significant between two groups, Bonferroni correction applied for three or more groups.

**Supplementary Table B:** Healthiness of school food menus based on the presence of a Healthy Food and Drink Policy or perceived barriers.

|  | | **Green (%)** | **Amber (%)** | **Red (%)** |
| --- | --- | --- | --- | --- |
| Total sample (n=80) | | 16.4 [2.8, 28.8] | 34.7 [8.4, 49.1] | 36.8 [23.7, 59.6] |
| Healthy Food and Drink policy | Yes | 16.7 [2.8, 30.9] | 34.7 [8.4, 49.1] | 36.8 [23.5, 57.7] |
|  | No^#^ | 17.7 [3.3, 28.8] | 37.4 [7.9, 65.9] | 33.8 [19.9, 61.5] |
| Barriers^ | Yes | 9.7 [2.8, 28.8] | 35.1 [9.4, 59.3] | 40.4 [23.5, 67.9] |
|  | No barriers | 25.0 [0.0, 32.3] | 34.4 [8.1, 46.8] | 37.5 [26.7, 43.1] |

Values reported as median [25^th^, 75^th^ percentile]
# including those who would like to have a food policy but currently do not.
**^** A total of 51/80 schools listed at least one barrier to providing healthy food and drinks in schools and are represented in the ‘yes’ category.
* p value <0.05 considered significant.
